# The Epidemiology of colorectal cancer in Guangzhou, China: A cross-sectional and age-period-cohort study

**DOI:** 10.1371/journal.pgph.0006287

**Published:** 2026-06-01

**Authors:** Di Wu, Ke Tang, Yinru Liang, Huan Xu, Yuanyuan Chen, Suixiang Wang, Dedong Wang, Boheng Liang, Lei Yang

**Affiliations:** 1 School of Public Health, Guangzhou Medical University. Institute of Public Health, Guangzhou Medical University & Guangzhou Center for Disease Control and Prevention, Guangzhou, Guangdong, China; 2 Department of non-Communicable Disease Control and Prevention, Guangzhou Center for Disease Control and Prevention, Guangzhou, Guangdong, China; 3 Shantou Chaoyang District People’s Hospital, Shantou, Guangdong, China; Mercer University School of Medicine, UNITED STATES OF AMERICA

## Abstract

To explore the epidemiological features and trends of colorectal cancer (CRC) incidence and mortality in Guangzhou, and offering scientific evidence for its prevention and control. Cross-sectional study with Joinpoint regression analysis was utilized to analyze CRC incidence and mortality trends. The age-period-cohort model was applied to evaluate the impacts of age, period, and birth cohort on CRC incidence and mortality. 36086 CRC cases and 15557 deaths were reported, 2011–2020. The age-standardized incidence rate increased from 23.20/100,000 in 2011 to 27.10/100,000 in 2020 (AAPC = 1.90%, 95% CI: 1.04% - 2.77%, P < 0.01), with significant rises in both the 0–49 age group and those aged 50 and above. The age-standardized mortality rate changed from 8.97/ 100,000 in 2011 to 9.55/100,000 in 2020, showing a stable, non - significant trend. The 5-year survival rate for CRC patients in Guangzhou was 46.27%. The APC model showed that CRC incidence and mortality generally increased with age. The period effect had an initial rise then a decline in risk, and CRC incidence risk increased with birth cohort while mortality risk first rose, peaked in the late 1950s, and then decreased. During 2011–2020, CRC incidence in Guangzhou showed an upward trend, while mortality was stable. Men had higher incidence and mortality than women. Rates were higher in those aged 50 and above, and there was a rising trend among those under 50. Promoting CRC prevention awareness, enhancing early diagnosis, and advocating healthy lifestyles are crucial.

## 1. Introduction

Colorectal cancer (CRC) ranks as the third most prevalent malignancy and the second leading cause of cancer mortality worldwide. Global Cancer Observatory (GLOBOCAN) 2020 estimates from the International Agency for Research on Cancer (IARC) indicate that CRC accounted for approximately 1.88 million incident cases and 0.9 million deaths annually, underscoring its substantial burden on global public health [1]. While CRC incidence and mortality rates have plateaued or even declined in high-income countries, they persist at the highest absolute levels worldwide. Conversely, low- and middle-income countries are witnessing a concerning upward trend in both incidence and mortality rates [2]. Nations experiencing rapid socioeconomic development typically demonstrate a steady increase in colorectal cancer incidence rates, with epidemiological trends closely paralleling improvements in the Human Development Index (HDI) [3,4]. Li et al.‘s longitudinal analysis of colorectal cancer epidemiology in China (1990–2019) demonstrated a concerning 30-year upward trajectory in both incidence and mortality rates, creating mounting pressures on the nation’s healthcare infrastructure [5]. United Nations Development Program (UNDP) data from 2016 identified Guangzhou, the provincial capital of Guangdong, as China’s top-performing city in terms of Human Development Index (HDI) achievement. Guangzhou’s rapid economic transformation has precipitated marked lifestyle changes among its residents, characterized by accelerated living pace and Westernized dietary shifts. These sociobehavioral transitions have coincided with rising incidence of colorectal cancer - now recognized as one of the most prevalent malignancies imposing a growing public health burden in this metropolitan region [6]. Epidemiological evidence demonstrates that while Guangzhou’s crude colorectal cancer incidence rates showed marked increases between 2000–2011, age-standardized incidence rates remained relatively stable during this period, suggesting demographic aging as a key contributing factor [7]. This study examines the epidemiological profile of colorectal cancer in Guangzhou by analyzing incidence and mortality data from 2011 to 2020. Through this temporal analysis, we aim to: (1) characterize contemporary trends in CRC burden, and (2) generate evidence to inform targeted prevention strategies and clinical management approaches for this metropolitan population.

## 2. Materials and methods

### 2.1. Data source

Colorectal cancer incidence data were systematically extracted from the Guangzhou Center for Disease Control and Prevention’s (GZCDC) population-based Cancer Registry Management System on 03/06/2024, including all malignant tumor cases classified under ICD-10 codes C18-C20 (colon, rectosigmoid junction, and rectum cancers). Mortality data were obtained from the GZCDC’s Cause of Death Registration System on 03/06/2024, which records underlying causes of death according to WHO standards. The analysis included 36,086 incident CRC cases and 15,557 CRC-attributable deaths occurring between 01/012011–31/12/2020. For each case, we collected demographic and clinical variables including gender, age, residence, date of birth, date of death, date of diagnosis, and survival status. Annual population denominators were derived from official residential registration data provided by the Guangzhou Public Security Bureau. These were cross-validated with census data to ensure accuracy. The crude incidence rate (CIR), crude mortality rate (CMR), age-standardized incidence rate (ASIR) and age-standardized mortality rate (ASMR) of colorectal cancer in Guangzhou from 2011 to 2020 were calculated based on the annual average population of Guangzhou and the age composition of Segi’s world standard population [8]. This study was approved by the ethics committee of Guangzhou Center for Disease Control and Prevention (The approval code of the ethics committee is GZCDC-ECHR-2023P0050).

### 2.2. Quality control of the data

Data quality control and validation procedures were rigorously performed in compliance with both national and international standards, specifically: (1) the 2016 Chinese Guidelines for Cancer Registration, and (2) the established quality assurance protocols of the International Agency for Research on Cancer (IARC) and the International Association of Cancer Registries (IACR) [9–11]. To evaluate data quality, we employed established cancer registry quality indicators including: (1) morphological verification rate (MV%), (2) death certificate-only cases (DCO%), and (3) mortality-to-incidence ratio (M/I). During the 2011–2020 study period, Guangzhou’s colorectal cancer registry demonstrated excellent data quality, with an M/I ratio of 0.43, high MV% of 87.31%, and minimal DCO% of 0.84%. These metrics collectively confirm the dataset’s completeness and reliability for epidemiological analysis. As a retrospective study using fully anonymized registry data, this research was exempt from institutional review board approval per standard protocols.

### 2.3. Statistical analyses

#### 2.3.1. Survival analysis.

Survival analysis was performed using the Kaplan-Meier method to estimate cumulative survival rates and median survival time. Between-group comparisons were assessed through Log-Rank tests, with statistical significance defined a priori as *P* < 0.05 (two-tailed)

#### 2.3.2. Joinpoint regression analysis.

The Joinpoint regression model was employed to identify significant temporal trends by partitioning the study period into distinct linear segments at statistically determined inflection points. This approach enables both comprehensive trend assessment and piecewise linear regression analysis for each identified interval. The optimal number and location of inflection points were determined through an iterative grid search algorithm (GSM), which systematically evaluates all possible segment combinations to maximize model fit while controlling for multiple comparisons [12]. Model selection was guided by the Bayesian Information Criterion (BIC), with optimal model determination based on minimal mean squared error (MSE) values. The final fitted model generated three key epidemiological metrics: (1) annual percentage change (APC) for each temporal segment, (2) average annual percent change (AAPC) for the entire study period, and (3) corresponding 95% confidence intervals (CIs). Using age-standardized incidence and mortality rates (ASIR and ASMR), we analyzed population-wide colorectal cancer trends in Guangzhou (2011–2020) across all age strata. The directionality of trends was interpreted as follows: positive APC values (with 95% CI excluding zero) indicated statistically significant increasing trends, while negative values denoted decreasing trends. All analyses were conducted using the Joinpoint Regression Program (Version 4.9.1.0, National Cancer Institute), with statistical significance threshold set at α = 0.05 (two-tailed).

#### 2.3.3. Age-Period-Cohort (APC) model analysis.

The Age-Period-Cohort (APC) model represents a well-established analytical framework in cancer epidemiology that disentangles three temporal dimensions: (1) age effects (biological aging processes), (2) period effects (environmental influences affecting all age groups simultaneously), and (3) cohort effects (exposures specific to birth cohorts). In this investigation, we implemented the APC model through the web-based analytical tool developed by the U.S. National Cancer Institute (NCI), which employs constrained generalized linear models (GLMs) with Poisson distribution to estimate these distinct temporal effects on colorectal cancer incidence patterns (https://analysistools.cancer.gov/apc/) [13]. Historically, 5-year age and calendar periods were commonly employed in age-period-cohort analyses, likely due to the prevalence of incidence rates reported in 5-year age intervals. However, utilizing shorter birth cohort intervals can more effectively delineate birth cohort risk patterns [14]. In this study’s age-period-cohort model, the age range was restricted to 30–99 years, with each age group encompassing a 2-year interval, resulting in 35 distinct groups (e.g., 30–31 years to 98–99 years). The diagnosis period was categorized into 5 groups (2011–2012–2019–2020), and the birth cohorts were divided into 39 groups (1912–1913–1988–1989). The reference groups for analysis were designated as the central age group, central period group, and central cohort group. The outcome measures include longitudinal age curves (expected age-specific rates in the reference period adjusted for cohort effects), period rate ratios (the ratio of age-specific rates in each period relative to the reference period), and cohort rate ratios (the ratios of specific age rates in each cohort relative to a reference cohort), which represent the age effect, period effect, and cohort effect, respectively.

## 3. Results

### 3.1. Overall situation of colorectal cancer and trends of ASIR and ASMR in Guangzhou from 2011 to 2020

Between 2011 and 2020, a total of 36,086 new cases of colorectal cancer were registered in Guangzhou. Of these, 20,549 cases (56.94%) occurred in males, while 15,537 cases (43.06%) were reported in females. In terms of mortality, during the same period, 15,557 individuals succumbed to colorectal cancer, with 8,860 cases (56.95%) in males and 6,697 cases (43.05%) in females.

**Fig 1:**
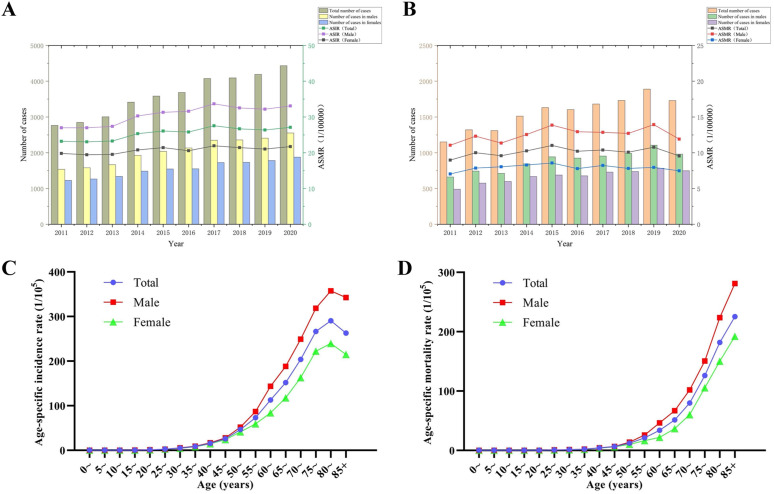
The incidence and mortality of colorectal cancer in Guangzhou, 2011-2020. **(A-B)** Age-standardized incidence/mortality rates and incidence/mortality numbers of colorectal cancer in Guangzhou from 2011 to 2020; **(C-D)** Age-specific incidence/mortality rates.

As illustrated in Table 1, the crude incidence rate (CIR) of colorectal cancer in Guangzhou from 2011 to 2020 was 41.04 per 100,000 individuals, while the age-standardized incidence rate (ASIR) was 27.43 per 100,000 individuals. The year 2017 recorded the highest ASIR. Regarding gender disparities, the ASIR for males was 32.99 per 100,000, approximately 1.5 times that of females, which was 22.38 per 100,000. The incidence numbers and ASIR for the entire population across different years are depicted in Fig 1A. Furthermore, as indicated in Table 2, the overall crude mortality rate (CMR) of colorectal cancer was reported as 17.69 per 100,000 individuals, with rates of 20.09 per 100,000 for males and 15.29 per 100,000 for females. The age-standardized mortality rate (ASMR) for the population was 10.11 per 100,000 individuals. Notably, the ASMR for males was 12.61 per 100,000, approximately 1.6 times that of females, which was 7.90 per 100,000. The standardized mortality rates and mortality numbers for the entire population in various years are presented in Fig 1B.

**Table 1:** The incidence rates of colorectal cancer by gender in Guangzhou, 2011-2020.

	Male				Female				Total			
Year	N	Population (million)	CIRs (1/10^5^)	ASIRs (1/10^5^)	N	Population (million)	CIRs (1/10^5^)	ASIRs (1/10^5^)	N	Population (million)	CIRs (1/10^5^)	ASIRs (1/10^5^)
2011	1536	4.12	37.05	26.98	1227	4.01	30.26	19.80	2763	8.13	33.70	23.20
2012	1581	4.15	37.75	26.98	1267	4.05	30.83	19.43	2848	8.20	34.32	23.05
2013	1666	4.19	39.38	27.36	1338	4.11	32.10	19.54	3004	8.30	35.76	23.27
2014	1928	4.23	44.88	30.30	1486	4.17	34.98	20.80	3414	8.40	39.95	25.33
2015	2037	4.30	46.65	31.29	1547	4.25	35.66	21.43	3584	8.55	41.17	26.08
2016	2133	4.37	47.46	31.59	1552	4.34	34.61	20.56	3685	8.71	41.04	25.8
2017	2349	4.49	50.75	33.68	1725	4.48	37.11	21.94	4074	8.97	43.92	27.53
2018	2358	4.63	49.65	32.52	1734	4.65	36.22	21.43	4092	9.28	42.91	26.69
2019	2407	4.75	49.20	32.19	1783	4.79	35.96	21.04	4190	9.54	42.53	26.36
2020	2554	4.89	52.20	33.08	1878	4.96	37.87	21.72	4432	9.85	44.99	27.10
Total	20549	44.12	46.59	32.99	15537	43.81	35.46	22.38	36086	87.93	41.04	27.43

CIRs crude incidence rate; ASIRs Age-standardized incidence rate.

**Table 2:** The mortality rates of colorectal cancer by gender in Guangzhou, 2011-2020.

Year	Male				Female				Total			
	N	Population (million)	CMRs (1/10^5^)	ASMRs (1/10^5^)	N	Population (million)	CMRs (1/10^5^)	ASMRs (1/10^5^)	N	Population (million)	CMRs (1/10^5^)	ASMRs (1/10^5^)
2011	661	4.12	16.06	11.05	490	4.01	12.21	7.03	1151	8.13	14.16	8.97
2012	744	4.15	17.95	12.31	576	4.05	14.21	7.85	1320	8.20	16.10	10.00
2013	712	4.19	17.00	11.35	598	4.11	14.55	8.04	1310	8.30	15.79	9.59
2014	844	4.23	19.95	12.54	668	4.17	16.03	8.28	1512	8.40	18.00	10.26
2015	943	4.30	21.95	13.86	688	4.25	16.19	8.57	1631	8.55	19.09	11.02
2016	925	4.37	21.18	12.94	678	4.34	15.63	7.78	1603	8.71	18.41	10.21
2017	953	4.49	21.21	12.85	729	4.48	16.25	8.22	1682	8.97	18.73	10.38
2018	992	4.63	21.43	12.70	738	4.65	15.88	7.81	1730	9.28	18.65	10.08
2019	1106	4.75	23.29	13.93	783	4.79	16.35	7.95	1889	9.54	19.81	10.78
2020	980	4.89	20.03	11.91	749	4.96	15.11	7.47	1729	9.85	17.55	9.55
Total	8860	44.12	20.09	12.61	6697	43.81	15.29	7.90	15557	87.93	17.69	10.11

CMRs crude mortality rate; ASMRs Age-standardized mortality rate.

Fig 1C and 1D show that when considering age, the incidence and mortality rates of colorectal cancer patients of different genders are relatively similar before the age of 50, with a relatively slow increase. After the age of 50, there is a more pronounced change in the incidence and mortality rates of colorectal cancer patients of different genders, with male colorectal cancer patients having higher incidence and mortality rates than female patients.

### 3.2. Age distribution, regional distribution and survival analysis

**Fig 2:**
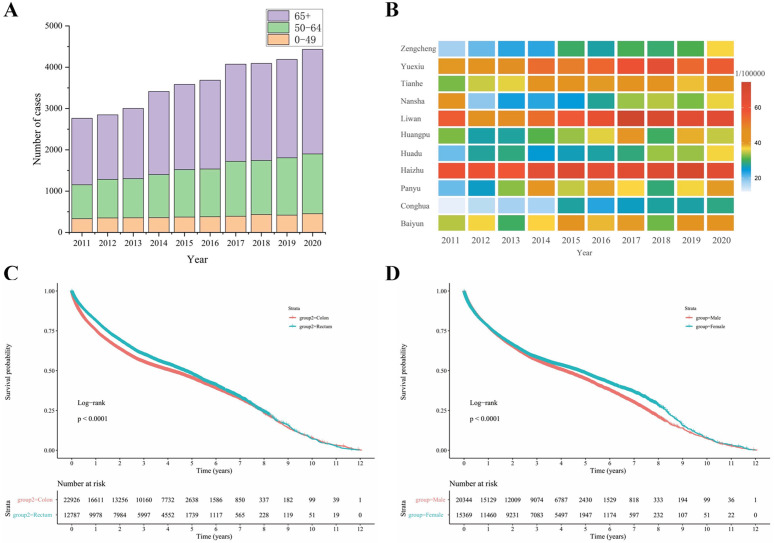
Age distribution, regional distribution and survival analysis of colorectal cancer patients in Guangzhou from 2011 to 2020. **(A)** Age distribution of colorectal cancer from 2011 to 2020; **(B)** Regional distribution of the colorectal cancer incidence population; **(C-D)** Survival curves of colorectal cancer patients with different pathogenic sites and different genders.

The median age of colorectal cancer cases reported in Guangzhou from 2011 to 2020 was 67 years. The study population was categorized into three age groups: under 49 years, 50–64 years, and over 64 years. A total of 3,840 cases of colorectal cancer were reported in individuals under 49 years (10.64%), 11,529 cases in the 50–64 age group (31.95%), and 20,717 cases in those aged 65 years and older (57.41%). The majority of cases were concentrated in individuals aged 50 years and above, accounting for 89.36% of the total. As illustrated in Fig 2A, from 2011 to 2020, the number of cases among those over 50 years of age has been increasing, particularly in the 50–64 age group.

Fig 2B illustrates the incidence of colorectal cancer by administrative region in Guangzhou from 2011 to 2020. The regions with the highest incidence rates were Liwan (6.28 per 100,000), Haizhu (6.26 per 100,000), and Yuexiu (5.61 per 100,000), respectively. In contrast, Conghua recorded the lowest incidence rate at 2.21 per 100,000.

The survival analysis results presented in Fig 2C and 2D indicate that the 5-year survival rate for colorectal cancer is 46.27%. More specifically, the 5-year survival rate for rectal cancer is 47.97%, while that for colon cancer is 45.32% (P < 0.001). Additionally, the 5-year survival rate is 48.39% for women and 44.66% for men (P < 0.001).

### 3.3. Joinpoint regression fitting results of ASIRs and ASMRs of colorectal cancer in Guangzhou, 2010 ~ 2020

**Fig 3:**
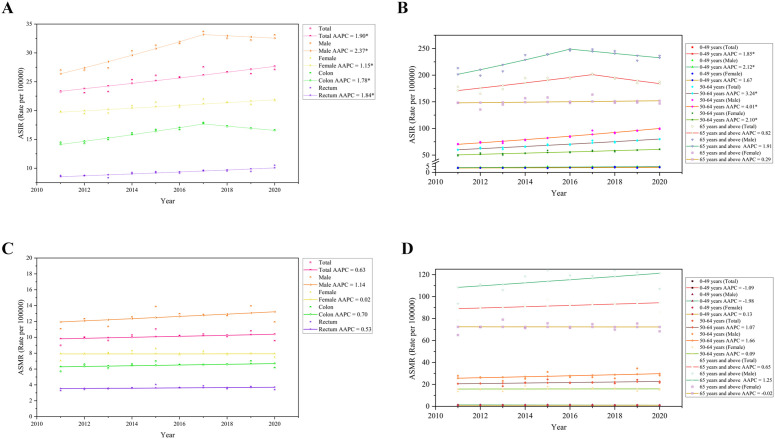
Time trend analysis of incidence and mortality of colorectal cancer in Guangzhou from 2011 to 2020. **(A)** Trends of age-standardized incidence of colorectal cancer (ASIR) in different sex and Site of disease in Guangzhou; **(B)** Trends of age-standardized incidence rates (ASIR) of colorectal cancer among different gender and age groups in Guangzhou; **(C)** Trends of age-standardized mortality of colorectal cancer (ASMR) in different sex and Site of disease in Guangzhou; **(D)** Trends of age-standardized mortality rates (ASMR) of colorectal cancer among different gender and age groups in Guangzhou.

The trend analysis of colorectal cancer incidence rates from 2011 to 2020, as illustrated in Fig 3A and Table 3, reveals an overall increase in the age-standardized incidence rate (ASIR) from 23.20 per 100,000 in 2011 to 27.10 per 100,000 in 2020 (AAPC = 1.90%, 95% CI: 1.04% to 2.77%, P < 0.01). Gender differences in incidence rates indicate a more rapid increase among males (AAPC = 2.37%, 95% CI: 1.01% to 3.74%, P < 0.01) compared to females (AAPC = 1.15%, 95% CI: 0.45% to 1.87%, P < 0.01). Notably, a significant inflection points in the male incidence rate occurred in 2017, marking the most substantial increase from 2010 to 2017 (APC = 3.90%, 95% CI: 2.21% to 5.61%, P < 0.01). When analyzing specific age groups and different sites of onset, the overall trends in the incidence and mortality rates of colorectal cancer in the colon (AAPC = 1.78%, 95% CI: 0.63% to 2.93%, P < 0.01) and rectum (AAPC = 1.84%, 95% CI: 0.90% to 2.80%, P < 0.01) exhibit similar upward trajectories. The incidence rate of colon cancer also demonstrated a significant inflection point in 2017, with the most pronounced increase observed prior to this year (APC = 3.81%, 95% CI: 2.39% to 5.24%, P < 0.01). Furthermore, as depicted in Fig 3B, the age group of 50–64 years experienced the most significant increase in colorectal cancer incidence rate (AAPC = 3.24%, 95% CI: 2.36% to 4.13%, P < 0.01), followed by the 0–49 age group (AAPC = 1.85%, 95% CI: 1.15% to 2.56%, P < 0.01).

**Table 3:** APC and AAPC of colorectal cancer age-standardized incidence rates by gender and age group in Guangzhou, 2011–2020.

Characteristics	Group	Year	APC (95% CI)	*t*	*p*	AAPC (95% CI)	*t*	*p*
All ages	Total	2011-2020	1.90*(1.04 ~ 2.77)	5.12	<0.01	1.90*(1.04 ~ 2.77)	5.12	<0.01
	Male	2011-2017	3.90*(2.21 ~ 5.61)	6.00	<0.01	2.37*(1.01 ~ 3.74)	3.43	<0.01
		2017-2020	-0.62 (-4.62 ~ 3.55)	-0.39	0.71			
	Female	2011-2020	1.15*(0.45 ~ 1.87)	3.77	<0.01	1.15*(0.45 ~ 1.87)	3.77	<0.01
Pathogenic site	Colon	2011-2017	3.81*(2.39 ~ 5.24)	6.99	<0.01	1.78*(0.63 ~ 2.93)	3.05	<0.01
		2017-2020	-2.16 (-5.53 ~ 1.32)	-1.60	0.17			
	Rectum	2011-2020	1.84*(0.90 ~ 2.80)	4.52	<0.01	1.84*(0.90 ~ 2.80)	4.52	<0.01
0-49 years	Total	2011-2020	1.85*(1.15 ~ 2.56)	6.08	<0.01	1.85*(1.15 ~ 2.56)	6.08	<0.01
	Male	2011-2020	2.12*(0.39 ~ 3.89)	2.83	<0.05	2.12*(0.39 ~ 3.89)	2.83	<0.05
	Female	2011-2020	1.67 (-0.66 ~ 4.05)	1.65	0.14	1.67 (-0.66 ~ 4.05)	1.65	0.14
50-64 years	Total	2011-2020	3.24*(2.36 ~ 4.13)	8.55	<0.01	3.24*(2.36 ~ 4.13)	8.55	<0.01
	Male	2011-2020	4.01*(3.03 ~ 5.00)	9.53	<0.01	4.01*(3.03 ~ 5.00)	9.53	<0.01
	Female	2011-2020	2.10*(1.05 ~ 3.17)	4.62	<0.01	2.10*(1.05 ~ 3.17)	4.62	<0.01
65 years and above	Total	2011-2017	2.72 (-0.07 ~ 5.59)	2.51	0.05	0.82 (-1.42 ~ 3.10)	0.71	0.48
		2017-2020	-2.89 (-9.34 ~ -4.03)	-1.10	0.32			
	Male	2011-2017	4.30*(0.05 ~ 8.75)	2.60	<0.05	1.61 (-0.83 ~ 4.11)	1.29	0.20
		2017-2020	-1.67 (-6.40 ~ 3.31)	-0.88	0.42			
	Female	2011-2020	0.29 (-1.07 ~ 1.67)	0.49	0.64	0.29 (-1.07 ~ 1.67)	0.49	0.64

APC annual percent change; AAPC average annual percent change

*the APC/AAPC is significantly different from zero at the alpha = 0.05 level

In terms of mortality trends (as illustrated in Fig 3C-3D and Table 4), the overall colorectal cancer mortality rate in Guangzhou exhibited a gradual increase from 2011 to 2020; however, this trend did not reach statistical significance (AAPC = 0.63%, 95% CI: -0.89% to 2.17%, P = 0.37). Among the various age groups, the 0–49 age group demonstrated a slow decline in colorectal cancer mortality rate (AAPC = -1.09%, 95% CI: -3.81% to 1.70%, P = 0.39), while the other age groups showed a gradual increase. Overall, the colorectal cancer mortality rate remained relatively stable, with no significant trend observed.

**Table 4:** APC and AAPC of colorectal cancer age-standardized mortality rates by gender and age group in Guangzhou, 2011–2020.

Characteristics	Group	Year	APC (95% CI)	*t*	*p*	AAPC (95% CI)	*p*
All ages	Total	2011-2020	0.63 (-0.89 ~ 2.17)	0.95	0.37	0.63 (-0.89 ~ 2.17)	0.37
	Male	2011-2020	1.14 (-0.69 ~ 2.99)	1.43	0.19	1.14 (-0.69 ~ 2.99)	0.19
	Female	2011-2020	0.01 (-1.42 ~ 1.48)	0.03	0.98	0.01 (-1.42 ~ 1.48)	0.98
Pathogenic site	Colon	2011-2020	0.70 (-0.83 ~ 2.25)	1.05	0.32	0.70 (-0.83 ~ 2.25)	0.32
	Rectum	2011-2020	0.53 (-1.21 ~ 2.29)	0.70	0.51	0.53 (-1.21 ~ 2.29)	0.51
0-49 years	Total	2011-2020	-1.09 (-3.81 ~ 1.70)	-0.91	0.39	-1.09 (-3.81 ~ 1.70)	0.39
	Male	2011-2020	-1.98 (-5.83 ~ 2.02)	-1.15	0.28	-1.98 (-5.83 ~ 2.02)	0.28
	Female	2011-2020	0.14 (-2.60 ~ 2.95)	0.11	0.91	0.13 (-2.60 ~ 2.95)	0.91
50-64 years	Total	2011-2020	1.07 (-0.96 ~ 3.14)	1.21	0.26	1.07 (-0.96 ~ 3.14)	0.26
	Male	2011-2020	1.66 (-1.30 ~ 4.72)	1.29	0.23	1.66 (-1.30 ~ 4.72)	0.23
	Female	2011-2020	0.09 (-2.94 ~ 3.22)	0.07	0.95	0.09 (-2.94 ~ 3.22)	0.95
65 years and above	Total	2011-2020	0.65 (-0.91 ~ 2.24)	0.96	0.37	0.65 (-0.91 ~ 2.24)	0.37
	Male	2011-2020	1.25 (-0.82 ~ 3.37)	1.39	0.20	1.25 (-0.82 ~ 3.37)	0.20
	Female	2011-2020	-0.02 (-1.48 ~ 1.47)	-0.03	0.98	-0.02 (-1.48 ~ 1.47)	0.98

APC annual percent change; AAPC average annual percent change

*the APC/AAPC is significantly different from zero at the alpha = 0.05 level

### 3.4. Age-period-cohort analysis

#### 3.4.1. Fitting of age-period-cohort model.

The results of the Age-Period-Cohort (APC) model regarding the incidence and mortality rates of colorectal cancer in Guangzhou from 2011 to 2020 demonstrate statistically significant age, period, and cohort deviations (all P < 0.05), as presented in S1 Table.

#### 3.4.2. Age-period-cohort analysis of colorectal cancer incidence.

**Fig 4:**
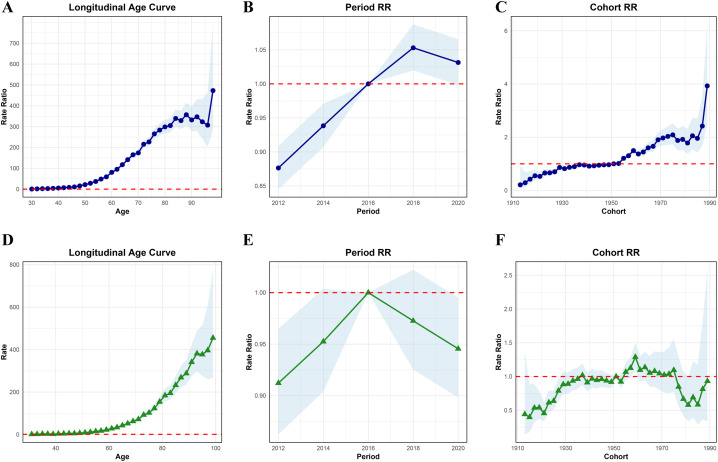
Age-period-cohort model analysis of colorectal cancer incidence and mortality in Guangzhou from 2011 to 2020. **(A-C)** Incidence: Longitudinal age curve, Period Rate Ratios, and Cohort Rate Ratios; **(D-F)** Mortality: Longitudinal age curve, Period Rate Ratios, and Cohort Rate Ratios. The shaded bands indicate 95% CIs; The dashed lines indicate the reference period (2015–2016 as reference) and cohort (1950–1951 birth cohort group as reference).

As illustrated in Fig 4A-4C and S2 Table, the longitudinal age-specific incidence rates of colorectal cancer in Guangzhou from 2011 to 2020 demonstrate an initial increase followed by a subsequent decrease. The peak incidence rate occurs within the 84–85 age group, reaching 340.19 cases per 100,000 individuals. Prior to this age group, the incidence rate of colorectal cancer continues to rise, after which a decline is observed post-84–85 years, followed by another increase until the 98–99 age group. The period effect results indicate that, with the shift of birth cohorts, the risk of colorectal cancer incidence initially rises before declining, peaking during the 2017–2018 period. The cohort effect reveals that, in comparison to the reference group born in 1950–1951, the risk of colorectal cancer increases more slowly for birth cohorts prior to this period than for those born afterward. Overall, individuals born later exhibit a higher risk of developing colorectal cancer.

#### 3.4.3. Age-period-cohort analysis of colorectal cancer mortality.

As illustrated in Fig 4D-4F and S2 Table, the age-related effects on colorectal cancer mortality rates in the population of Guangzhou from 2011 to 2020 reveal a consistent increase in mortality with advancing age. Specifically, the mortality rate escalates from 1.27 per 100,000 in the 30–31 age group to 455.34 per 100,000 in the 98–99 age group. The period effect analysis indicates that, using the 2015–2016 period as a reference, the risk of colorectal cancer mortality rose steadily prior to 2015–2016, followed by a subsequent decline. Furthermore, the cohort effect results illustrate a general trend of increasing mortality risk followed by a decrease. When using the birth cohort of 1950–1951 as a reference, the risk of colorectal cancer mortality in cohorts born before 1959 exhibits an overall increasing trend, peaking around 1959. Thereafter, the risk of mortality from colorectal cancer begins to decline and stabilize, before experiencing another upward trend.

## 4. Discussion

The results of this study indicate that from 2011 to 2020, the incidence rate of colorectal cancer in the population of Guangzhou has exhibited a consistent upward trend. Specifically, the overall crude incidence rate (CIR) of colorectal cancer rose from 33.70 per 100,000 in 2011 to 44.99 per 100,000 in 2020, while the age-standardized incidence rate (ASIR) increased from 23.20 per 100,000 in 2011 to 27.10 per 100,000 in 2020. In contrast, the mortality rate has remained relatively stable. Over the past decade, there has been a continuous rise in the number of colorectal cancer cases in Guangzhou, with the crude incidence rate demonstrating a steady upward trajectory [7]. In comparison, the latest estimates of colorectal cancer trends from the National Cancer Center of China in 2022 indicate that the standardized incidence rate of colorectal cancer in China was approximately 18.05 per 100,000 in 2016, while the standardized mortality rate was around 8.13 per 100,000 [15]. Currently, the incidence and mortality rates of colorectal cancer in Guangzhou surpass the national averages.

The survival analysis results indicate that the 5-year survival rate for colorectal cancer patients in Guangzhou from 2011 to 2020 was 46.27%, with higher survival rates reported for females compared to males, aligning with findings from other studies [16]. Furthermore, when stratified by site of onset, the survival rate for rectal cancer surpassed that of colon cancer. One study revealed that the overall 5-year survival rate for colorectal cancer patients in China rose from 47.2% in 2003–2005 to 56.9% in 2012–2015 [17]. In comparison, the 5-year survival rate for colorectal cancer in Guangzhou was lower than that observed in this study. A review of clinical staging data revealed that most patients were diagnosed at an advanced stage (Stage III and IV), with the exception of those with unclear clinical staging records. Additionally, a study based on data from the National Center for Health Statistics in the United States demonstrated that the 5-year relative survival rate for colorectal cancer increased from 50% in the mid-1970s to 65% in 2012–2018 [18]. This trend underscores the long-term benefits afforded to colorectal cancer patients through continuous advancements in early screening and treatment technologies [19,20]. Despite the improved treatment outcomes and increased 5-year survival rates for colorectal cancer in recent years, the rising incidence of colorectal cancer among younger individuals presents significant economic burdens and public health challenges. Therefore, promoting colorectal cancer screening and early diagnosis is essential for alleviating the disease burden on the population [21].

Through joinpoint regression analysis, we observed an overall increasing trend in the incidence rate of colorectal cancer in Guangzhou. When stratified by gender, a significant inflection point in the incidence rate for males was identified in 2017. Specifically, there was a clear upward trend in incidence rates from 2011 to 2017, followed by a gradual decline from 2017 to 2020. In contrast, the age-standardized mortality rate (ASMR) for colorectal cancer remained relatively stable. It has been reported that the colorectal cancer screening program in Guangzhou was officially launched in 2015 [22], with the first round of screening conducted from 2015 to 2017. During the early stages of screening, significant changes in the number of colorectal cancer cases were observed. We speculate that the rapid increase in the incidence rate of colorectal cancer among males prior to 2017 may be attributed to the impact of the screening program, reflecting its effectiveness. In terms of incidence rate trends across different genders, changes in colorectal cancer incidence rates were more pronounced in males than in females. This disparity may be linked to the increasing work and life pressures faced by young males, alongside the rising prevalence of risk factors such as alcohol consumption, smoking, high-fat diets, and obesity, all of which elevate the risk of developing colorectal cancer in this demographic [23–26]. Statistical analyses from multiple countries or regions indicate that males represent the primary population group for alcohol consumption [27,28], which has a more detrimental impact on male health compared to females. Furthermore, it has been established that alcohol consumption is directly associated with an increased risk of colorectal cancer [29,30].

The trend in incidence rates among different age groups in Guangzhou indicates that the population under 50 years old is experiencing an overall increase in colorectal cancer cases, reflecting a decrease in the average age of onset among patients. Notably, the 50–64 age group exhibits a rapid rise in incidence rates, while the most significant changes for those aged 65 and above occurred between 2011 and 2017. Data show that incidence and mortality rates of colorectal cancer increase sharply after the age of 50, with 90% of new cases and deaths globally occurring in individuals aged 50 and older [31]. The trend of younger onset colorectal cancer underscores the necessity to expand screening programs to include high-risk individuals under 50, while maintaining regular screenings for those aged 50 and above. Efforts to promote healthier lifestyles through increased public awareness are crucial. Furthermore, investigating the factors contributing to the younger onset of colorectal cancer and enhancing healthcare services for the elderly are essential for effectively managing and alleviating the disease burden.

The results of the age effect analysis indicate that as the population in Guangzhou ages, the incidence rate of colorectal cancer exhibits a trend of initially increasing and then subsequently decreasing. This finding aligns with a study conducted on European populations [32]; however, it is noteworthy that the peak age for colorectal cancer incidence in this study occurs around 85 years, which is slightly delayed compared to the peak incidence age of approximately 75 years observed in European populations. In the age group of 98–99 years, the sharp increase in incidence rate may be biased due to the extremely small number of cases. Furthermore, as age advances, the mortality rate of colorectal cancer also rises significantly. Given the increasing aging population, along with the burden of chronic underlying diseases and diminished immune function in the elderly, middle-aged and elderly individuals represent a crucial demographic for the prevention and treatment of colorectal cancer.

The results of the period effect analysis indicate that prior to 2017–2018, the incidence of colorectal cancer continued to rise, while the mortality risk associated with colorectal cancer increased until 2015–2016. This observation indirectly highlights the significant impact of the colorectal cancer screening program implemented in Guangzhou between 2015 and 2017 on both the incidence and mortality rates of colorectal cancer [33].

In the analysis of cohort effects, using the birth cohort of 1950–1951 as a reference, no significant changes were observed in the risk of colorectal cancer incidence prior to this period. However, as the birth cohorts advanced, the risk of colorectal cancer steadily increased. Notably, more recent birth cohorts have experienced a sharp rise in the risk of developing colorectal cancer. On one hand, improvements in living standards have led to gradual changes in dietary patterns [34,35], characterized by increased consumption of high-fat, oily, salty, sugary, and red meat foods [36–39]. This shift has significantly heightened the risk of developing colorectal cancer. On the other hand, the heightened work and mental stress associated with economic development have contributed to a notable rise in unhealthy lifestyles, including alcohol consumption, smoking, and sedentary behavior [40,41]. These factors are critical contributors to the increasing incidence rate of colorectal cancer.

Prior to the birth cohort of 1950–1951, particularly before 1938, there was a notable increase in the risk of colorectal cancer mortality. Variations in cohort effects may be linked to the social environment and healthcare levels in China. During the 1920s and 1930s, China experienced periods of war and social unrest, which resulted in poor living conditions and limited awareness of healthy diets among the population, consequently leading to an increased risk of disease-related mortality. However, following the establishment of the People’s Republic of China, social stability was achieved, living conditions improved, and significant advancements were made in health education and healthcare. Birth cohorts from later years had better dietary conditions compared to those from earlier years, resulting in a stabilization of the risk of colorectal cancer mortality. Notably, in the late 1950s, there was a peak in the mortality risk among colorectal cancer patients in the birth cohorts. Between 1959 and 1961, China faced one of the worst famines in human history, which had severe health and economic consequences for survivors, particularly those who experienced famine in early childhood, potentially contributing to the increased risk [42]. In recent years, improvements in social security, stable social conditions, positive economic development, and significant enhancements in healthcare have contributed to a sustained reduction in the risk of colorectal cancer mortality. Additionally, the gradual implementation of a series of cancer prevention and control measures may have also played a role in this reduction.

The strength of this study lies in its provision of an up-to-date assessment of time trends in colorectal cancer incidence and mortality in Guangzhou. The age-period-cohort model further elucidates the independent roles of age, period, and birth cohort, offering valuable insights into the analysis of long-term registry data and providing scientific evidence for cancer prevention and ongoing surveillance. However, this study also has certain limitations. First, our focus on trend analysis precludes the quantification of risk factors that may contribute to changes in colorectal cancer trends. Secondly, due to limitations in tumor registration, our data could not be updated to the most recent time. Additionally, the secondary development and implementation of screening technologies in recent years have also influenced trends in cancer incidence and mortality. Therefore, these factors should be considered and addressed in our follow-up research, as we continue to assess the long-term impact of these trends in the future.

## 5. Conclusions

In summary, from 2011 to 2020, the incidence of colorectal cancer in Guangzhou exhibited an overall upward trend. Influenced by factors such as the intensity of colorectal cancer screening, short-term trends indicate a continued increase in male incidence until 2017, followed by a subsequent decline. In contrast, the death rate remained relatively stable, with evidence of a slight gradual increase, but no distinct trend was observed. The incidence and mortality rates of colorectal cancer are higher in men compared to women. Furthermore, morbidity and mortality rates are elevated among individuals aged 50 years and older; however, the incidence of colorectal cancer in individuals under the age of 50 is also on the rise. Strengthening targeted prevention and treatment measures, particularly for men and those aged 50 years and older, will significantly alleviate the disease burden associated with colorectal cancer. Concurrently, enhancing colorectal cancer prevention strategies for the younger population is equally imperative.

## Supporting information

S1 Table The Wald Chi-Square test of Age-Period-Cohort model estimable function of colorectal cancer incidence and mortality in Guangzhou from 2011 to 2020. (DOCX)

S2 Table Age-Period-Cohort model analysis of colorectal cancer incidence and mortality in Guangzhou, 2011–2020. (DOCX)

## References

[1] Sung H, Ferlay J, Siegel RL. Global cancer statistics 2020: GLOBOCAN estimates of incidence and mortality worldwide for 36 cancers in 185 countries. CA Cancer J Clin. 2021;71(3):209–49. 33538338 10.3322/caac.21660

[2] Arnold M, Sierra MS, Laversanne M, Soerjomataram I, Jemal A, Bray F. Global patterns and trends in colorectal cancer incidence and mortality. Gut. 2017;66(4):683–91. doi: 10.1136/gutjnl-2015-310912 26818619

[3] Fidler MM, Soerjomataram I, Bray F. A global view on cancer incidence and national levels of the human development index. Int J Cancer. 2016;139(11):2436–46. doi: 10.1002/ijc.30382 27522007

[4] Lu B, Li N, Luo C-Y, Cai J, Lu M, Zhang Y-H, et al. Colorectal cancer incidence and mortality: the current status, temporal trends and their attributable risk factors in 60 countries in 2000-2019. Chin Med J (Engl). 2021;134(16):1941–51. doi: 10.1097/CM9.0000000000001619 34238851 PMC8382382

[5] Li Q, Wu H, Cao M, Li H, He S, Yang F, et al. Colorectal cancer burden, trends and risk factors in China: A review and comparison with the United States. Chin J Cancer Res. 2022;34(5):483–95. doi: 10.21147/j.issn.1000-9604.2022.05.08 36398126 PMC9646460

[6] Cao W, Chen H-D, Yu Y-W, Li N, Chen W-Q. Changing profiles of cancer burden worldwide and in China: a secondary analysis of the global cancer statistics 2020. Chin Med J (Engl). 2021;134(7):783–91. doi: 10.1097/CM9.0000000000001474 33734139 PMC8104205

[7] Zhou Q, Li K, Lin G-Z, Shen J-C, Dong H, Gu Y-T, et al. Incidence trends and age distribution of colorectal cancer by subsite in Guangzhou, 2000-2011. Chin J Cancer. 2015;34(8):358–64. doi: 10.1186/s40880-015-0026-6 26245843 PMC4593365

[8] Ahmad OB, Boschi-Pinto C, Lopez AD, Murray CJL, Lozano R, Inoue M. Age standardization of rates: a new WHO standard. 2000.

[9] Guo M, Xu J, Du J. Trends in cervical cancer mortality in China from 1989 to 2018: an age-period-cohort study and Joinpoint analysis. BMC Public Health. 2021;21(1):1329. doi: 10.1186/s12889-021-11401-8 34229639 PMC8259057

[10] Bray F, Parkin DM. Evaluation of data quality in the cancer registry: principles and methods. Part I: comparability, validity and timeliness. Eur J Cancer. 2009;45(5):747–55. doi: 10.1016/j.ejca.2008.11.032 19117750

[11] Parkin DM, Bray F. Evaluation of data quality in the cancer registry: principles and methods Part II. Completeness. Eur J Cancer. 2009;45(5):756–64. doi: 10.1016/j.ejca.2008.11.033 19128954

[12] Kim HJ, Fay MP, Feuer EJ, Midthune DN. Permutation tests for joinpoint regression with applications to cancer rates. Stat Med. 2000;19(3):335–51. doi: 10.1002/(sici)1097-0258(20000215)19:3<335::aid-sim336>3.0.co;2-z 10649300

[13] Rosenberg PS, Check DP, Anderson WF. A web tool for age-period-cohort analysis of cancer incidence and mortality rates. Cancer Epidemiol Biomarkers Prev. 2014;23(11):2296–302. doi: 10.1158/1055-9965.EPI-14-0300 25146089 PMC4221491

[14] Tarone RE, Chu KC. Evaluation of birth cohort patterns in population disease rates. Am J Epidemiol. 1996;143(1):85–91. doi: 10.1093/oxfordjournals.aje.a008661 8533751

[15] Zheng R, Zhang S, Zeng H, Wang S, Sun K, Chen R, et al. Cancer incidence and mortality in China, 2016. J Natl Cancer Cent. 2022;2(1):1–9. doi: 10.1016/j.jncc.2022.02.002 39035212 PMC11256658

[16] Zeng C, Wen W, Morgans AK, Pao W, Shu X-O, Zheng W. Disparities by Race, Age, and Sex in the Improvement of Survival for Major Cancers: Results From the National Cancer Institute Surveillance, Epidemiology, and End Results (SEER) Program in the United States, 1990 to 2010. JAMA Oncol. 2015;1(1):88–96. doi: 10.1001/jamaoncol.2014.161 26182310 PMC4523124

[17] Zeng H, Chen W, Zheng R, Zhang S, Ji JS, Zou X, et al. Changing cancer survival in China during 2003-15: a pooled analysis of 17 population-based cancer registries. Lancet Glob Health. 2018;6(5):e555–67. doi: 10.1016/S2214-109X(18)30127-X 29653628

[18] Siegel RL, Wagle NS, Cercek A, Smith RA, Jemal A. Colorectal cancer statistics, 2023. *CA Cancer J Clin* 2023; 73(3): 233–54. 10.3322/caac.21772 36856579

[19] Hillner BE, Siegel BA, Liu D, Shields AF, Gareen IF, Hanna L, et al. Impact of positron emission tomography/computed tomography and positron emission tomography (PET) alone on expected management of patients with cancer: initial results from the National Oncologic PET Registry. J Clin Oncol. 2008;26(13):2155–61. doi: 10.1200/JCO.2007.14.5631 18362365

[20] Gross CP, Andersen MS, Krumholz HM, McAvay GJ, Proctor D, Tinetti ME. Relation between Medicare screening reimbursement and stage at diagnosis for older patients with colon cancer. JAMA. 2006;296(23):2815–22. doi: 10.1001/jama.296.23.2815 17179458

[21] Campos FG. Colorectal cancer in young adults: A difficult challenge. World J Gastroenterol. 2017;23(28):5041–4. doi: 10.3748/wjg.v23.i28.5041 28811701 PMC5537173

[22] Li Y, Liu HZ, Liang YR, et al. Analysis of community colorectal cancer screening in 50-74 years old people in Guangzhou, 2015-2016. Zhonghua Liu Xing Bing Xue Za Zhi. 2018;39(1):81–5. 29374902 10.3760/cma.j.issn.0254-6450.2018.01.017

[23] Gausman V, Dornblaser D, Anand S. Risk Factors Associated With Early-Onset Colorectal Cancer. Clinical Gastroenterology and Hepatology: The Official Clinical Practice Journal of the American Gastroenterological Association. 2020;18(12):2752-9.e2. 10.1016/j.cgh.2019.10.009 PMC7153971 31622737

[24] Low EE, Demb J, Liu L, Earles A, Bustamante R, Williams CD, et al. Risk Factors for Early-Onset Colorectal Cancer. Gastroenterology. 2020;159(2):492-501.e7. doi: 10.1053/j.gastro.2020.01.004 31926997 PMC7343609

[25] Chang VC, Cotterchio M, De P, Tinmouth J. Risk factors for early-onset colorectal cancer: a population-based case-control study in Ontario, Canada. Cancer Causes Control. 2021;32(10):1063–83. doi: 10.1007/s10552-021-01456-8 34120288 PMC8416813

[26] Karastergiou K, Smith SR, Greenberg AS, Fried SK. Sex differences in human adipose tissues - the biology of pear shape. Biol Sex Differ. 2012;3(1):13. doi: 10.1186/2042-6410-3-13 22651247 PMC3411490

[27] Wilsnack RW, Wilsnack SC, Kristjanson AF, Vogeltanz-Holm ND, Gmel G. Gender and alcohol consumption: patterns from the multinational GENACIS project. Addiction. 2009;104(9):1487–500. doi: 10.1111/j.1360-0443.2009.02696.x 19686518 PMC2844334

[28] The global burden of disease attributable to alcohol and drug use in 195 countries and territories, 1990-2016: a systematic analysis for the Global Burden of Disease Study 2016. The Lancet Psychiatry. 2018;5(12):987–1012. 30392731 10.1016/S2215-0366(18)30337-7 PMC6251968

[29] Garaycoechea JI, Crossan GP, Langevin F, Mulderrig L, Louzada S, Yang F, et al. Alcohol and endogenous aldehydes damage chromosomes and mutate stem cells. Nature. 2018;553(7687):171–7. doi: 10.1038/nature25154 29323295 PMC6047743

[30] Im PK, Yang L, Kartsonaki C, Chen Y, Guo Y, Du H, et al. Alcohol metabolism genes and risks of site-specific cancers in Chinese adults: An 11-year prospective study. Int J Cancer. 2022;150(10):1627–39. doi: 10.1002/ijc.33917 35048370 PMC7612513

[31] Keum N, Giovannucci E. Global burden of colorectal cancer: emerging trends, risk factors and prevention strategies. Nat Rev Gastroenterol Hepatol. 2019;16(12):713–32. doi: 10.1038/s41575-019-0189-8 31455888

[32] Long D, Mao C, Zhang Z, Liu Y, Li J, Xu Y, et al. Long-term trends in the burden of colorectal cancer in Europe over three decades: a joinpoint regression and age-period-cohort analysis. Front Oncol. 2023;13:1287653. doi: 10.3389/fonc.2023.1287653 38115907 PMC10728819

[33] Fang Y, Xiao B, Peng J, Tian H, Wang F, Kong L, et al. An early report of a screening program for colorectal cancer in Guangzhou, China. Ann Transl Med. 2019;7(21):604. doi: 10.21037/atm.2019.09.75 32047765 PMC7011592

[34] Deng Y, Wei B, Zhai Z. Dietary risk-related colorectal cancer burden: estimates from 1990 to 2019. Frontiers in Nutrition. 2021;8:690663. 34504859 10.3389/fnut.2021.690663 PMC8421520

[35] Yu W, Jiang J, Xie L. Mortality trends in colorectal cancer in China during 2000-2015: A joinpoint regression and age-period-cohort analysis. Preventing Chronic Disease. 2018;15:E156. 10.5888/pcd15.180329 PMC6307832 30576278

[36] Zheng X, Hur J, Nguyen LH, et al. Comprehensive Assessment of Diet Quality and Risk of Precursors of Early-Onset Colorectal Cancer. *Journal of the* National Cancer Institute 2021; 113(5): 543–52. 10.1093/jnci/djaa164 PMC8096368 33136160

[37] Khan NA, Hussain M, ur Rahman A, Farooqui WA, Rasheed A, Memon AS. Dietary Practices, Addictive Behavior and Bowel Habits and Risk of Early Onset Colorectal Cancer: a Case Control Study. Asian Pac J Cancer Prev. 2015;16(17):7967–73. doi: 10.7314/apjcp.2015.16.17.7967 26625827

[38] Archambault AN, Lin Y, Jeon J, et al. Nongenetic Determinants of Risk for Early-Onset Colorectal Cancer. JNCI cancer spectrum 2021; 5(3). 10.1093/jncics/pkab029 PMC8134523 34041438

[39] Hur J, Otegbeye E, Joh H-K, Nimptsch K, Ng K, Ogino S, et al. Sugar-sweetened beverage intake in adulthood and adolescence and risk of early-onset colorectal cancer among women. Gut. 2021;70(12):2330–6. doi: 10.1136/gutjnl-2020-323450 33958435 PMC8571123

[40] Yu J, Feng Q, Kim JH, Zhu Y. Combined Effect of Healthy Lifestyle Factors and Risks of Colorectal Adenoma, Colorectal Cancer, and Colorectal Cancer Mortality: Systematic Review and Meta-Analysis. Front Oncol. 2022;12:827019. doi: 10.3389/fonc.2022.827019 35936678 PMC9353059

[41] Boyle T, Heyworth J, Bull F, McKerracher S, Platell C, Fritschi L. Timing and intensity of recreational physical activity and the risk of subsite-specific colorectal cancer. Cancer Causes Control. 2011;22(12):1647–58. doi: 10.1007/s10552-011-9841-5 21922204

[42] Chen Y, Zhou L-A. The long-term health and economic consequences of the 1959-1961 famine in China. J Health Econ. 2007;26(4):659–81. doi: 10.1016/j.jhealeco.2006.12.006 17289187

