# Supplementary material for: The Epidemiology of colorectal cancer in Guangzhou, China: A cross-sectional and age-period-cohort study
Source: PLOS Glob Public Health. 2026 Jun 1;6(6):e0006287. doi: 10.1371/journal.pgph.0006287 (PMC13225427; doi:10.1371/journal.pgph.0006287)
Supplement: S1 Table — (DOCX) [file pgph.0006287.s001.docx]

S1 Table. The Wald Chi-Square test of Age-Period-Cohort model estimable function of colorectal cancer incidence and mortality in Guangzhou from 2011 to 2020

| Null hypothesis | Implications | Degrees of freedom | Incidence | | Mortality | |
| --- | --- | --- | --- | --- | --- | --- |
|  |  |  | *χ*^2^ | *P* value | *χ*^2^ | *P* value |
| Net drift = 0 | Fitted temporal trends are stable (i.e., flat with no change) over time.  Fitted longitudinal and cross-sectional age curves are proportional. | 1 | 98.64 | <0.01 | 1.60 | 0.21 |
| All age deviations = 0 | Fitted longitudinal and cross-sectional age curves are log-linear (i.e., log-additive). | 33 | 471.79 | <0.01 | 62.40 | <0.01 |
| All period deviations = 0 | Fitted temporal trends and period rate ratios are log-linear (i.e., log-additive). | 3 | 23.38 | <0.01 | 11.32 | <0.05 |
| All cohort deviations = 0 | Cohort rate ratios are log-linear; all local drifts equal the net drift. | 37 | 129.59 | <0.01 | 78.28 | <0.01 |
| All period rate ratios = 1 | Net drift is 0 and fitted temporal trends are constant;  Cross-sectional age curve describes age incidence pattern in every period. | 4 | 114.58 | <0.01 | 12.25 | <0.01 |
| All cohort rate ratios = 1 | Net drift is 0 and all local drifts are 0;  Longitudinal age curve describes age incidence pattern in every cohort. | 38 | 239.07 | <0.01 | 89.98 | <0.01 |
| All local drifts = the net drift | Temporal patterns are the same in every age group. | 35 | 125.56 | <0.01 | 76.20 | <0.01 |
