# Supplementary material for: The Epidemiology of colorectal cancer in Guangzhou, China: A cross-sectional and age-period-cohort study
Source: PLOS Glob Public Health. 2026 Jun 1;6(6):e0006287. doi: 10.1371/journal.pgph.0006287 (PMC13225427; doi:10.1371/journal.pgph.0006287)
Supplement: S2 Table — (DOCX) [file pgph.0006287.s002.docx]

S2 Table. Age-Period-Cohort model analysis of colorectal cancer incidence and mortality in Guangzhou, 2011-2020

| project | incidence | |  | mortality | |
| --- | --- | --- | --- | --- | --- |
|  | Rate/Rate Ratio | 95%CI |  | Rate/Rate Ratio | 95%CI |
| Age (year) |  |  |  |  |  |
| 30-31 | 1.55 | (1.10,2.19) |  | 1.27 | (0.61,2.65) |
| 32-33 | 2.35 | (1.74,3.16) |  | 1.59 | (0.82,3.08) |
| 34-35 | 3.22 | (2.46,4.20) |  | 2.91 | (1.74,4.88) |
| 36-37 | 3.69 | (2.90,4.69) |  | 2.88 | (1.83,4.53) |
| 38-39 | 4.80 | (3.87,5.95) |  | 2.04 | (1.31,3.18) |
| 40-41 | 6.19 | (5.10,7.51) |  | 3.66 | (2.57,5.23) |
| 42-43 | 7.76 | (6.53,9.22) |  | 4.22 | (3.05,5.85) |
| 44-45 | 9.36 | (7.99,10.98) |  | 5.18 | (3.85,6.98) |
| 46-47 | 11.77 | (10.16,13.63) |  | 5.24 | (3.97,6.92) |
| 48-49 | 16.48 | (14.47,18.78) |  | 7.03 | (5.51,8.97) |
| 50-51 | 22.55 | (20.01,25.40) |  | 8.97 | (7.22,11.13) |
| 52-53 | 29.21 | (26.20,32.57) |  | 11.46 | (9.43,13.93) |
| 54-55 | 37.81 | (34.30,41.68) |  | 15.08 | (12.67,17.95) |
| 56-57 | 49.48 | (45.33,54.00) |  | 17.48 | (14.96,20.43) |
| 58-59 | 60.07 | (55.44,65.10) |  | 22.22 | (19.29,25.59) |
| 60-61 | 81.48 | (76.09,87.25) |  | 28.18 | (25.01,31.76) |
| 62-63 | 96.34 | (90.43,102.63) |  | 33.58 | (30.10,37.46) |
| 64-65 | 118.36 | (111.45,125.69) |  | 42.91 | (38.74,47.54) |
| 66-67 | 142.46 | (134.33,151.08) |  | 51.91 | (46.92,57.42) |
| 68-69 | 165.83 | (155.86,176.43) |  | 62.41 | (56.26,69.23) |
| 70-71 | 175.05 | (162.20,188.92) |  | 71.88 | (63.37,81.54) |
| 72-73 | 216.41 | (199.32,234.98) |  | 92.63 | (81.03,105.90) |
| 74-75 | 227.67 | (208.09,249.10) |  | 102.47 | (88.74,118.33) |
| 76-77 | 266.25 | (241.99,292.95) |  | 123.27 | (105.97,143.40) |
| 78-79 | 284.43 | (257.11,314.64) |  | 154.07 | (131.73,180.19) |
| 80-81 | 299.71 | (269.42,333.41) |  | 184.05 | (156.54,216.40) |
| 82-83 | 306.00 | (273.34,342.56) |  | 195.13 | (164.93,230.86) |
| 84-85 | 340.19 | (301.44,383.93) |  | 232.57 | (195.13,277.20) |
| 86-87 | 329.89 | (288.66,377.02) |  | 268.72 | (223.34,323.32) |
| 88-89 | 358.04 | (308.46,415.58) |  | 287.95 | (235.62,351.90) |
| 90-91 | 333.23 | (279.52,397.26) |  | 341.15 | (274.63,423.79) |
| 92-93 | 348.09 | (280.22,432.38) |  | 381.46 | (297.25,489.52) |
| 94-95 | 324.14 | (243.13,432.16) |  | 377.15 | (276.37,514.69) |
| 96-97 | 308.13 | (204.24,464.89) |  | 396.36 | (260.31,603.52) |
| 98-99 | 474.04 | (283.88,791.58) |  | 455.34 | (266.90,776.82) |
| Period (year) |  |  |  |  |  |
| 2011-2012 | 0.88 | (0.85，0.91) |  | 0.91 | (0.86，0.96) |
| 2013-2014 | 0.94 | (0.91，0.97) |  | 0.95 | (0.90，1.00) |
| 2015-2016 | 1.00 | (1.00，1.00) |  | 1.00 | (1.00，1.00) |
| 2017-2018 | 1.05 | (1.02，1.09) |  | 0.97 | (0.93，1.02) |
| 2019-2020 | 1.03 | (1.00，1.07) |  | 0.95 | (0.90，1.00) |
| Cohort (year) |  |  |  |  |  |
| 1912-1913 | 0.21 | (0.05,0.92) |  | 0.44 | (0.14,1.37) |
| 1914-1915 | 0.29 | (0.12,0.69) |  | 0.40 | (0.18,0.89) |
| 1916-1917 | 0.42 | (0.25,0.72) |  | 0.54 | (0.32,0.89) |
| 1918-1919 | 0.55 | (0.38,0.79) |  | 0.54 | (0.37,0.78) |
| 1920-1921 | 0.53 | (0.40,0.70) |  | 0.46 | (0.33,0.62) |
| 1922-1923 | 0.66 | (0.53,0.81) |  | 0.61 | (0.48,0.79) |
| 1924-1925 | 0.66 | (0.56,0.79) |  | 0.64 | (0.51,0.80) |
| 1926-1927 | 0.70 | (0.60,0.81) |  | 0.79 | (0.65,0.96) |
| 1928-1929 | 0.86 | (0.76,0.99) |  | 0.88 | (0.73,1.07) |
| 1930-1931 | 0.82 | (0.72,0.92) |  | 0.89 | (0.75,1.06) |
| 1932-1933 | 0.87 | (0.77,0.97) |  | 0.94 | (0.79,1.11) |
| 1934-1935 | 0.89 | (0.80,0.99) |  | 0.96 | (0.82,1.13) |
| 1936-1937 | 0.96 | (0.87,1.07) |  | 1.02 | (0.87,1.19) |
| 1938-1939 | 0.95 | (0.87,1.05) |  | 0.91 | (0.78,1.06) |
| 1940-1941 | 0.91 | (0.83,1.00) |  | 0.97 | (0.84,1.12) |
| 1942-1943 | 0.92 | (0.85,1.01) |  | 0.95 | (0.83,1.09) |
| 1944-1945 | 0.95 | (0.88,1.02) |  | 0.96 | (0.85,1.09) |
| 1946-1947 | 0.96 | (0.90,1.03) |  | 0.94 | (0.84,1.06) |
| 1948-1949 | 0.97 | (0.91,1.03) |  | 0.92 | (0.82,1.03) |
| 1950-1951 | 1.00 | (1.00,1.00) |  | 1.00 | (1.00,1.00) |
| 1952-1953 | 1.01 | (0.95,1.08) |  | 0.92 | (0.82,1.03) |
| 1954-1955 | 1.21 | (1.13,1.29) |  | 1.07 | (0.95,1.20) |
| 1956-1957 | 1.30 | (1.21,1.41) |  | 1.13 | (0.99,1.29) |
| 1958-1959 | 1.50 | (1.37,1.63) |  | 1.29 | (1.11,1.50) |
| 1960-1961 | 1.37 | (1.24,1.51) |  | 1.10 | (0.92,1.31) |
| 1962-1963 | 1.44 | (1.30,1.60) |  | 1.14 | (0.95,1.37) |
| 1964-1965 | 1.60 | (1.43,1.80) |  | 1.05 | (0.85,1.30) |
| 1966-1967 | 1.66 | (1.46,1.89) |  | 1.08 | (0.85,1.37) |
| 1968-1969 | 1.90 | (1.66,2.18) |  | 1.05 | (0.80,1.36) |
| 1970-1971 | 1.97 | (1.69,2.30) |  | 1.02 | (0.76,1.37) |
| 1972-1973 | 2.03 | (1.72,2.41) |  | 1.04 | (0.75,1.43) |
| 1974-1975 | 2.09 | (1.73,2.52) |  | 1.10 | (0.77,1.56) |
| 1976-1977 | 1.88 | (1.51,2.33) |  | 0.85 | (0.56,1.29) |
| 1978-1979 | 1.92 | (1.52,2.42) |  | 0.67 | (0.42,1.07) |
| 1980-1981 | 1.78 | (1.37,2.31) |  | 0.58 | (0.34,1.00) |
| 1982-1983 | 2.05 | (1.55,2.73) |  | 0.69 | (0.39,1.22) |
| 1984-1985 | 1.96 | (1.41,2.71) |  | 0.58 | (0.29,1.18) |
| 1986-1987 | 2.42 | (1.66,3.52) |  | 0.81 | (0.36,1.82) |
| 1988-1989 | 3.93 | (2.48,6.24) |  | 0.93 | (0.34,2.58) |
